# Supplementary material for: Taxonomic Characterization and Secondary Metabolite Analysis of NEAU-wh3-1: An Embleya Strain with Antitumor and Antibacterial Activity
Source: Microorganisms. 2020 Mar 20;8(3):441. doi: 10.3390/microorganisms8030441 (PMC7143961; doi:10.3390/microorganisms8030441)
Supplement: Supplementary file 1 [file microorganisms-08-00441-s001.pdf]

Taxonomic Characterization and Secondary Metabolite Analysis of NEAU-wh3-1: an *Embleya* strain with Antitumor and Antibacterial Activity

Han Wang [1,†], Tianyu Sun [1,†], Wenshuai Song[1], Xiaowei Guo [1], Peng Cao [1], Xi Xu [1], Yue Shen [1,2*], Junwei Zhao [1,*]

[1] Key Laboratory of Agricultural Microbiology of Heilongjiang Province, Northeast Agricultural University, No. 600 Changjiang Road, Xiangfang District, Harbin 150030, China; wanghan507555536@gmail.com (H.W.); sty1561214024@163.com (T.S.); wenshuaisong@163.com (W.S.); guoweizi@hotmail.com (X.G.); cp511@126.com (P.C.); xuxi1758899581@126.com

[2] College of Science, Northeast Agricultural University, No. 600 Changjiang Road, Xiangfang District, Harbin 150030, China

* Correspondence: shenyuelele@163.com (Y.S.); guyan2080@126.com (J.Z.)

† These authors contributed equally to this work.

Table S1. GenBank accession numbers of the sequences used in MLSA.

Strain	Type strain	Whole genome	*atp*D	*gyr*B	*rec*A	*rpo*B	*trp*B
Streptomyces radiciphilus	NEAU-wh3-1	–	MT039411	MT039412	MT039413	MT039414	MT039415
Streptomyces hyalinus	MB891-A1^T	BIFH01000000	–	–	–	–	–
Embleya scabrispora	DSM 41855^T	MWQN00000000	–	–	–	–	–
Streptomyces qinglanensis	172205^T	LJGV01000022	–	–	–	–	–
Streptomyces glebosus	CGMCC 4.1873^T	BLIO00000000	–	–	–	–	–
Streptomyces durbertensis	NEAU-S1GS20^T	–	MH534864	MH534865	MH534866	MH534867	MH534868
Streptomyces ramulosus	NRRL B-2714^T	–	MF581785	MF188173	MF581787	MF581789	MF581783
Streptomyces barkulensis	RC 1831^T	PGSG00000000	–	–	–	–	–
Streptacidiphilus bronchialis	DSM 106435^T	CP031264	–	–	–	–	–
Kitasatospora xanthocidica	NBRC 13469^T	QVIG00000000	–	–	–	–	–
Streptomyces griseoplanus	NRRL B-3064^T	LIQR00000000	KT384582	KT384931	JQ806239	JQ806243	JQ806247
Streptomyces cattleya	NRRL 8057^T	JWDF00000000	KT384775	KT385123	KT385477	KT389095	KT389444
Streptomyces rubrisoli	FXJ1.725^T	–	KC137297	KC137293	KC137289	KC137285	KC137281
Streptomyces rubidus	13C15^T	MDCQ00000000	KT384713	KT385063	KT385414	KT389034	KT389382
Streptomyces niger	NBRC 13362^T	JOFQ00000000	FJ406130	FJ406186	FJ406242	FJ406298	FJ406353
Streptomyces sparsogenes	ATCC 25498^T	ASQP00000000	KT384724	KT385073	KT385425	DQ241995	KT389393
Streptomyces hiroshimensis	NBRC 3839^T	–	KT384596	KT384945	KT385294	AY280780	KT389265
Streptomyces mobaraensis	NBRC 13819^T	VOKX00000000	KT384648	KT384997	KT385348	KT388968	KT389317
Streptomyces abikoensis	NBRC 13860^T	–	FJ406150	FJ406206	FJ406262	FJ406318	FJ406373
Streptomyces sclerotialus	NRRL ISP-5269^T	JOBC00000000	FJ406181	FJ406237	FJ406293	FJ406348	FJ406404
Streptomyces pratens	BK 138^T	–	KP890254	KP890257	KP890260	KP890263	KP890266
Streptomyces monomycini	NRRL B-24309^T	JNYL00000000	FJ406180	KT384998	KT385349	FJ406347	FJ406403
Streptomyces rimosus subsp. *rimosus*	ATCC 10970^T	LGCS00000000	FJ406141	FJ406197	FJ406253	KJ996628	FJ406364
Streptomyces olivaceiscleroticus	DSM 40595^T	–	FJ406164	FJ406220	FJ406276	FJ406332	FJ406387
Streptomyces catenulae	NRRL B-2342^T	JODY00000000	FJ406152	FJ406208	FJ406264	KJ996525	FJ406375
Streptomyces sioyaensis	NRRL B-5408^T	SDIF00000000	MG881213	FJ406188	MG881215	MG881217	MG881219
Streptomyces decoyicus	NRRL 2666^T	LGUU00000000	FJ406159	FJ406215	FJ406271	FJ406327	FJ406382
Streptomyces caniferus	NBRC 15389^T	BLIN00000000	KT384499	KT384848	KT385196	KT388818	KT389168
Streptomyces libani subsp. *rufus*	LMG 20087^T	BLIQ00000000	FJ406165	FJ406221	FJ406277	FJ406333	FJ406388
Streptomyces hygroscopicus subsp. *glebosus*	NBRC 13786^T	–	FJ406158	FJ406214	FJ406270	FJ406326	FJ406381
Streptomyces platensis	JCM 4662^T	MIGA00000000	GU383329	FJ406219	KJ469294	GU383753	KT389356

Table S2. MLSA distance values for selected strains in this study.

Strains: 1, NEAU-wh3-1; 2, *Streptomyces platensis* JCM 4662^{T}; 3, *Streptomyces hygroscopicus* subsp. *glebosus* NBRC 13786^{T}; 4, *Streptomyces glebosus* CGMCC 4.1873^{T}; 5, *Streptomyces libani* subsp. *rufus* LMG 20087^{T}; 6, *Streptomyces ramulosus* NRRL B-2714^{T}; 7, *Streptomyces caniferus* NBRC 15389^{T}; 8, *Streptomyces decoyicus* NRRL 2666^{T}; 9, *Streptomyces catenulae* NRRL B-2342^{T}; 10, *Streptomyces sioyaensis* NRRL B-5408^{T}; 11, *Streptomyces pratens* BK 138^{T}; 12, *Streptomyces abikoensis* NBRC 13860^{T}; 13, *Streptomyces hiroshimensis* NBRC 3839^{T}; 14, *Streptomyces sparsogenes* ATCC 25498^{T}; 15, *Streptomyces mobaraensis* NBRC 13819^{T}; 16, *Streptomyces rimosus* subsp. *rimosus* ATCC 10970^{T}; 17, *Streptomyces sclerotialus* NRRL ISP-5269^{T}; 18, *Streptomyces monomycini* NRRL B-24309^{T}; 19, *Streptomyces olivaceiscleroticus* DSM 40595^{T}; 20, *Streptomyces niger* NBRC 13362^{T}; 21, *Streptomyces durbertensis* NEAU-S1GS20^{T}; 22, *Streptomyces rubrisoli* FXJ1.725^{T}; 23, *Streptomyces qinglanensis* 172205^{T}; 24, *Streptomyces rubidus* 13C15^{T}; 25, *Kitasatospora xanthocidica* NBRC 13469^{T}; 26, *Streptacidiphilus bronchialis* DSM 106435^{T}; 27, *Streptomyces griseoplanus* NRRL B-3064^{T}; 28, *Streptomyces cattleya* NRRL 8057^{T}; 29, *Streptomyces barkulensis* RC 1831^{T}; 30, *Embleya hyalina* MB891-A1^{T}; 31, *Embleya scabrispora* DSM 41855^{T}.

Strain	MLSA (Kimura 2-parameter) distance																													
	1	2	3	4	5	6	7	8	9	10	11	12	13	14	15	16	17	18	19	20	21	22	23	24	25	26	27	28	29	30
1																														
2	0.526																													
3	0.472	0.053																												
4	0.412	0.558	0.490																											
5	0.481	0.077	0.030	0.505																										
6	0.565	0.389	0.369	0.670	0.370																									
7	0.464	0.097	0.047	0.522	0.067	0.375																								
8	0.464	0.085	0.040	0.522	0.061	0.375	0.043																							
9	0.470	0.129	0.079	0.564	0.100	0.316	0.081	0.073																						
10	0.475	0.112	0.063	0.533	0.087	0.371	0.064	0.067	0.088																					
11	0.477	0.165	0.132	0.561	0.144	0.380	0.136	0.135	0.136	0.131																				
12	0.464	0.168	0.129	0.574	0.126	0.365	0.115	0.126	0.106	0.123	0.138																			
13	0.513	0.178	0.168	0.633	0.161	0.376	0.168	0.173	0.155	0.175	0.187	0.116																		
14	0.536	0.204	0.193	0.673	0.180	0.416	0.183	0.195	0.203	0.197	0.215	0.170	0.109																	
15	0.458	0.161	0.120	0.556	0.116	0.366	0.114	0.117	0.107	0.111	0.117	0.076	0.133	0.168																
16	0.459	0.135	0.096	0.554	0.116	0.352	0.092	0.087	0.081	0.104	0.123	0.120	0.168	0.188	0.117															
17	0.475	0.162	0.123	0.577	0.137	0.393	0.116	0.123	0.130	0.112	0.091	0.132	0.173	0.204	0.125	0.118														
18	0.475	0.133	0.097	0.560	0.117	0.352	0.094	0.089	0.087	0.105	0.131	0.118	0.171	0.183	0.121	0.026	0.128													
19	0.456	0.137	0.098	0.559	0.117	0.361	0.095	0.092	0.080	0.094	0.134	0.110	0.159	0.190	0.120	0.085	0.112	0.089												
20	0.478	0.180	0.144	0.581	0.146	0.395	0.138	0.146	0.150	0.142	0.158	0.135	0.185	0.207	0.124	0.141	0.155	0.144	0.153											
21	0.508	0.355	0.308	0.620	0.313	0.120	0.307	0.309	0.327	0.311	0.329	0.330	0.398	0.412	0.316	0.319	0.331	0.319	0.330	0.325										
22	0.471	0.180	0.139	0.587	0.132	0.400	0.129	0.135	0.138	0.139	0.147	0.114	0.176	0.188	0.118	0.135	0.153	0.141	0.133	0.125	0.349									
23	0.640	0.941	0.885	0.327	0.907	1.020	0.901	0.912	0.907	0.896	0.932	0.929	0.962	0.995	0.914	0.919	0.945	0.922	0.938	0.956	0.979	0.933								
24	0.459	0.179	0.145	0.570	0.144	0.381	0.135	0.143	0.136	0.140	0.134	0.118	0.165	0.196	0.107	0.129	0.141	0.128	0.142	0.118	0.321	0.131	0.927							
25	0.715	0.384	0.366	0.968	0.378	0.675	0.363	0.366	0.354	0.345	0.360	0.369	0.384	0.387	0.360	0.365	0.367	0.356	0.364	0.383	0.661	0.373	0.749	0.375						
26	0.700	0.376	0.357	0.963	0.372	0.674	0.358	0.363	0.355	0.345	0.349	0.350	0.364	0.366	0.339	0.354	0.354	0.346	0.353	0.371	0.655	0.363	0.739	0.367	0.105					
27	0.449	0.186	0.150	0.579	0.161	0.408	0.154	0.159	0.152	0.152	0.159	0.150	0.210	0.225	0.146	0.145	0.162	0.146	0.152	0.159	0.362	0.159	0.957	0.144	0.363	0.311				
28	0.460	0.167	0.125	0.564	0.126	0.368	0.120	0.127	0.122	0.129	0.143	0.109	0.164	0.184	0.122	0.115	0.131	0.121	0.120	0.116	0.317	0.097	0.916	0.116	0.361	0.350	0.146			
29	0.527	0.289	0.272	0.554	0.268	0.305	0.274	0.282	0.273	0.275	0.273	0.262	0.268	0.282	0.253	0.259	0.278	0.267	0.272	0.240	0.357	0.265	0.861	0.234	0.515	0.524	0.299	0.245		
30	0.186	0.519	0.499	0.392	0.503	0.555	0.493	0.508	0.502	0.508	0.508	0.495	0.510	0.526	0.492	0.498	0.517	0.507	0.495	0.521	0.536	0.515	0.457	0.499	0.614	0.607	0.496	0.489	0.475	
31	0.195	0.524	0.505	0.483	0.513	0.567	0.503	0.512	0.498	0.493	0.512	0.497	0.513	0.526	0.486	0.505	0.515	0.508	0.500	0.520	0.551	0.522	0.548	0.507	0.607	0.605	0.498	0.493	0.545	0.108

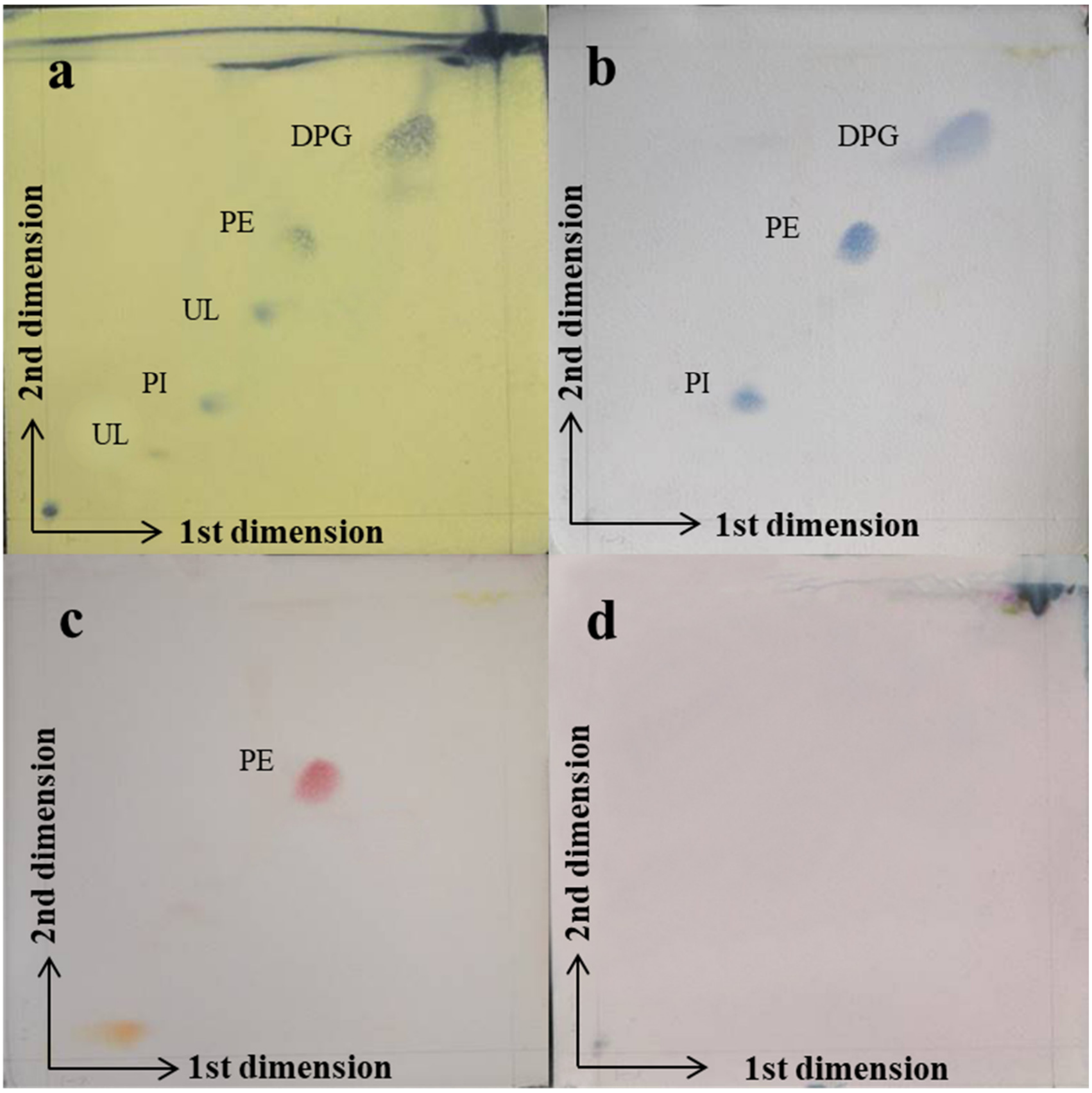

Figure 1. The polar lipids of strain NEAU-wh3-1. a, using molybdophosphoric acid reagent; b, molybdenum blue reagent; c, using ninhydrin reagent, d, using anisaldehyde reagent. Diphosphatidylglycerol (DPG), phosphatidylethanolamine (PE), phosphatidylinositol (PI), and unidentified lipid (UL); 1st dimension: chloroform: methanol: water (65: 25: 4, v/v); 2nd dimension: chloroform: acetic acid: methanol: water (80: 18: 12: 5, v/v).

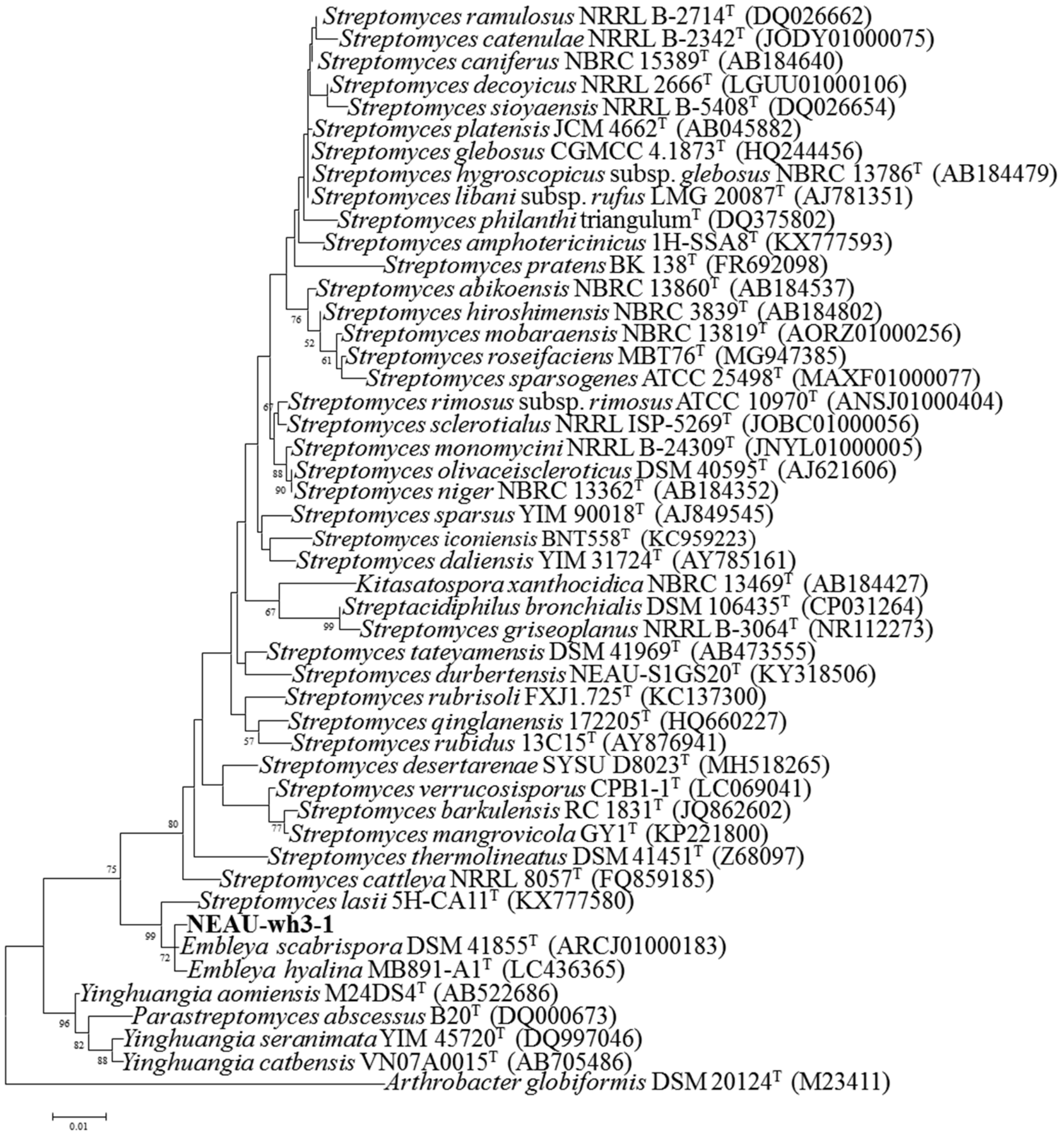

Figure S2. Maximum-likelihood tree based on 16S rRNA gene sequences showing relationship between strain NEAU-wh3-1 and related taxa based on 16S rRNA gene sequences. Only bootstrap values above 50 % (percentages of 1000 replications) are indicated. *Arthrobacter globiformis* DSM 20124^{T} (M23411) was used as an outgroup. Bar, 0.01 substitutions per nucleotide position.

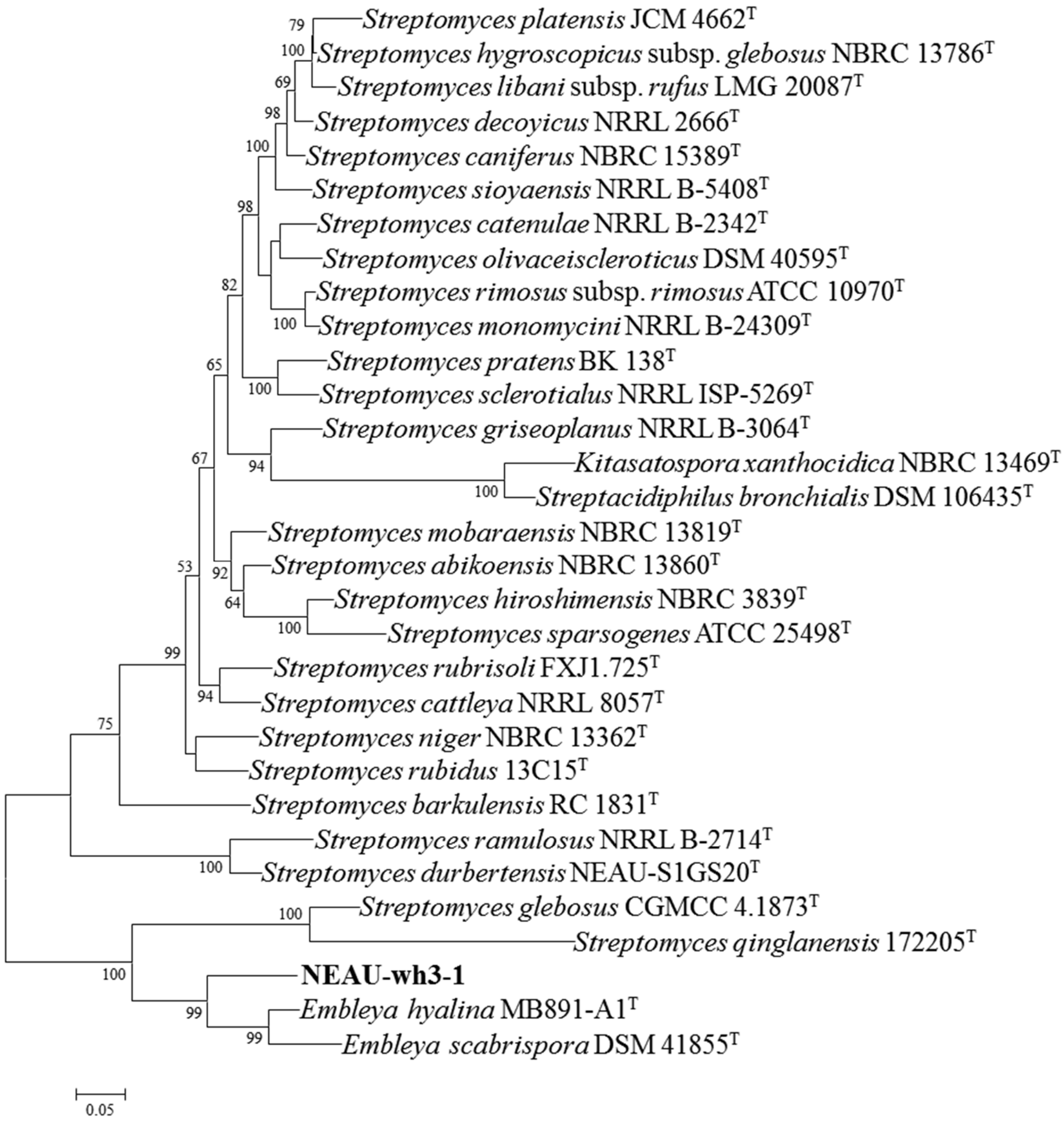

Figure S3 Maximum-likelihood tree based on multilocus sequence analysis (MLSA) analysis of the concatenated partial sequences (1979 bp) from five housekeeping genes (*atp*D, *gyr*B, *rec*A, *rpo*B, and *trp*B) of strain NEAU-wh3-1 (in bold) with related taxa. Only bootstrap values above 50% (percentages of 1000 replications) are indicated. Bar, 0.05 substitutions per nucleotide position.

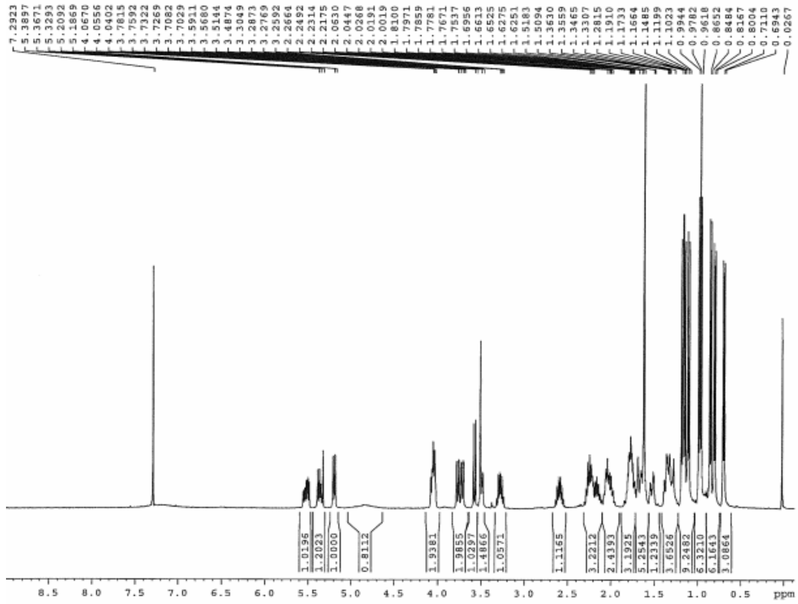

Figure. S4. ^{1}H NMR (400 MHz) spectrum of compound **1** in $CDCl_3$

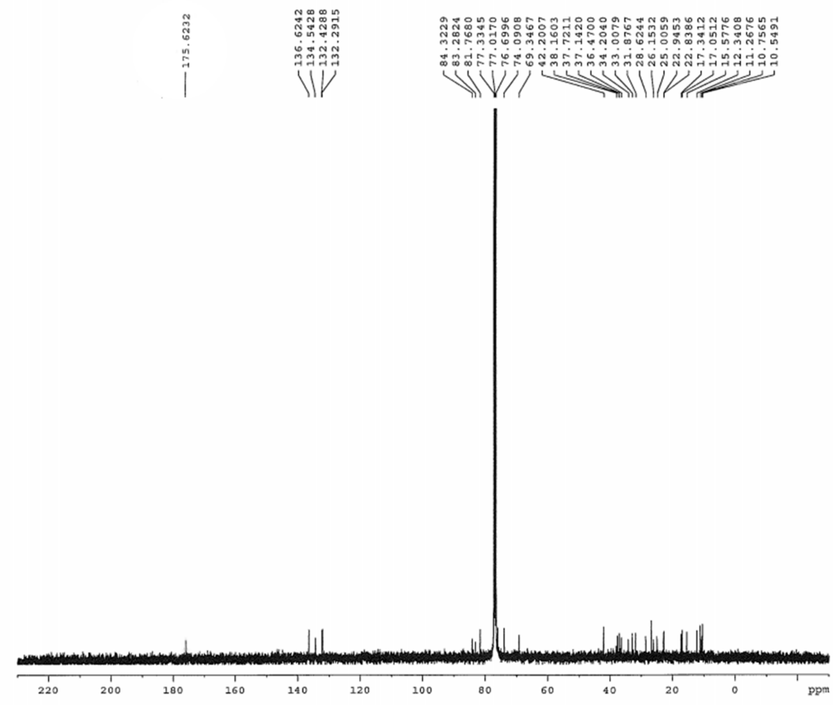

Figure S5. ^{13}C NMR (150 MHz) spectrum of compound **1 (**in $CDCl_3$)

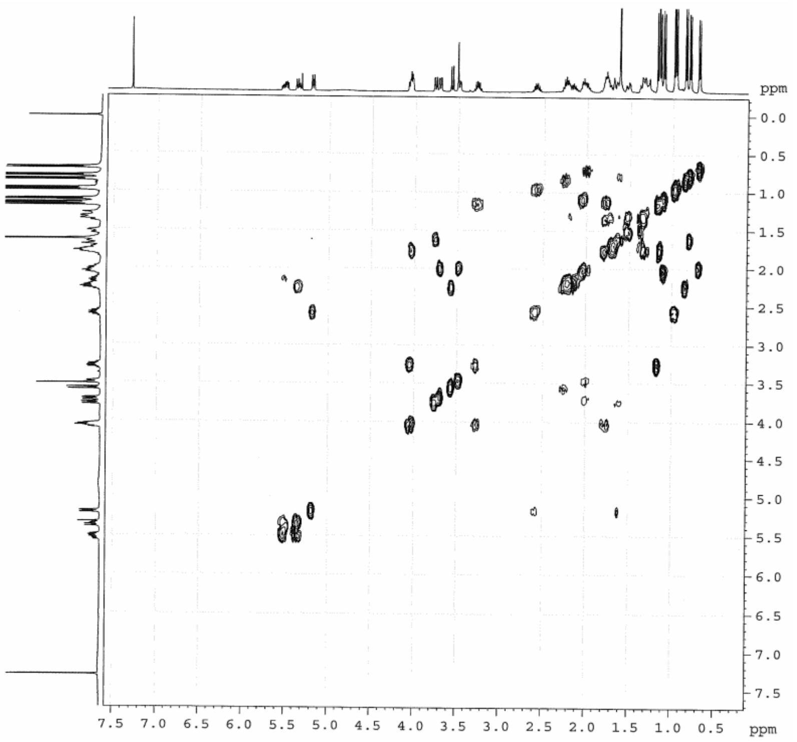

Figure S6. ^{1}H-^{1}H COSY spectrum (400 MHz) of compound **1** (in $CDCl_3$)

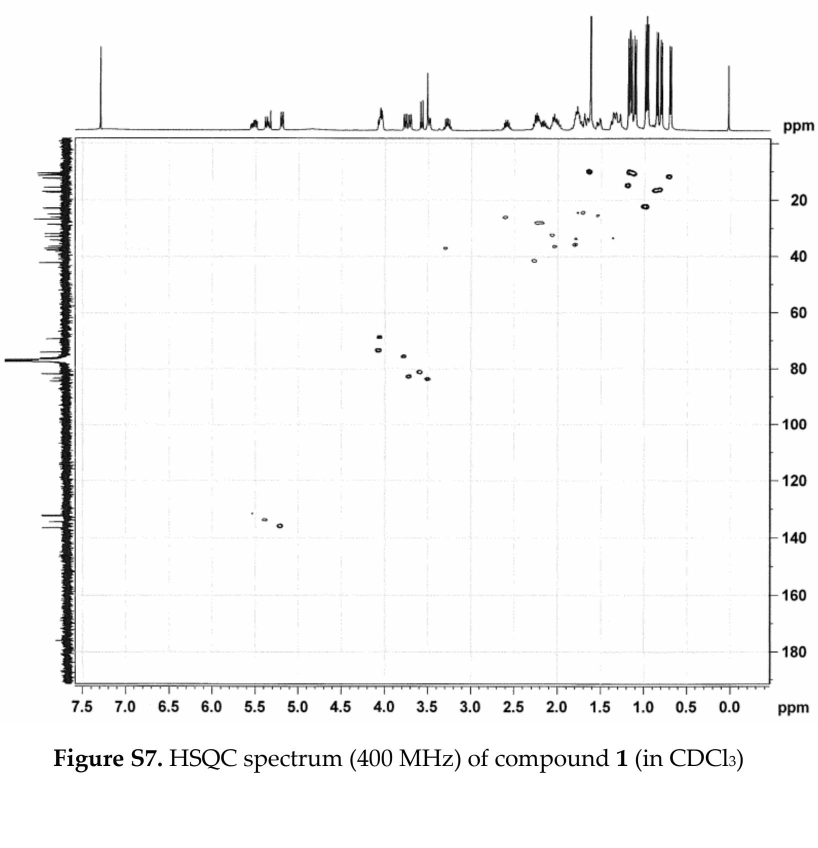

Figure S7. HSQC spectrum (400 MHz) of compound **1** (in $CDCl_3$)

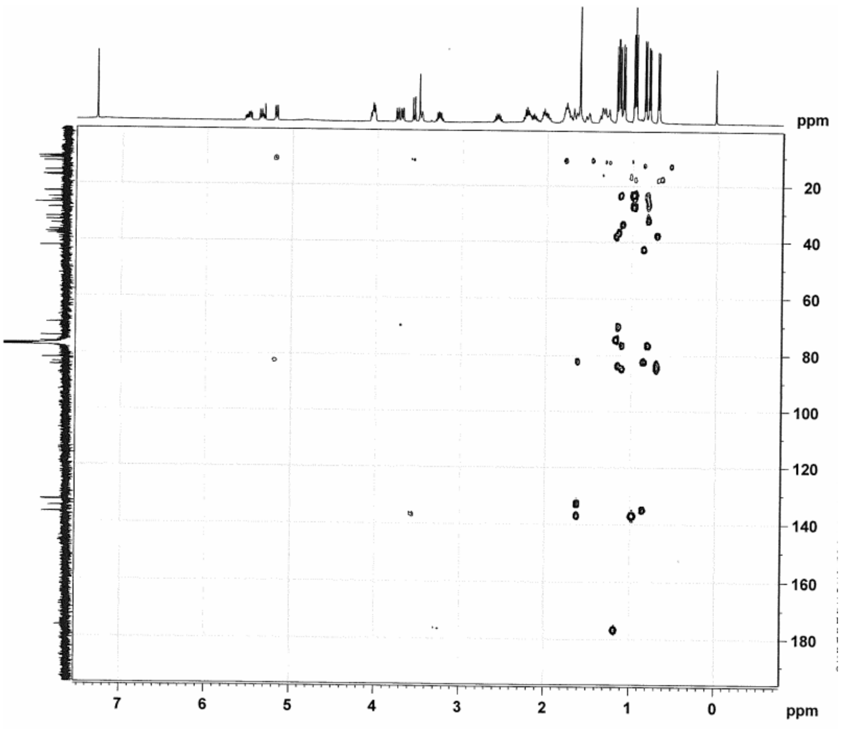

Figure S8. HMBC spectrum (400 MHz) of compound **1** (in $CDCl_3$)

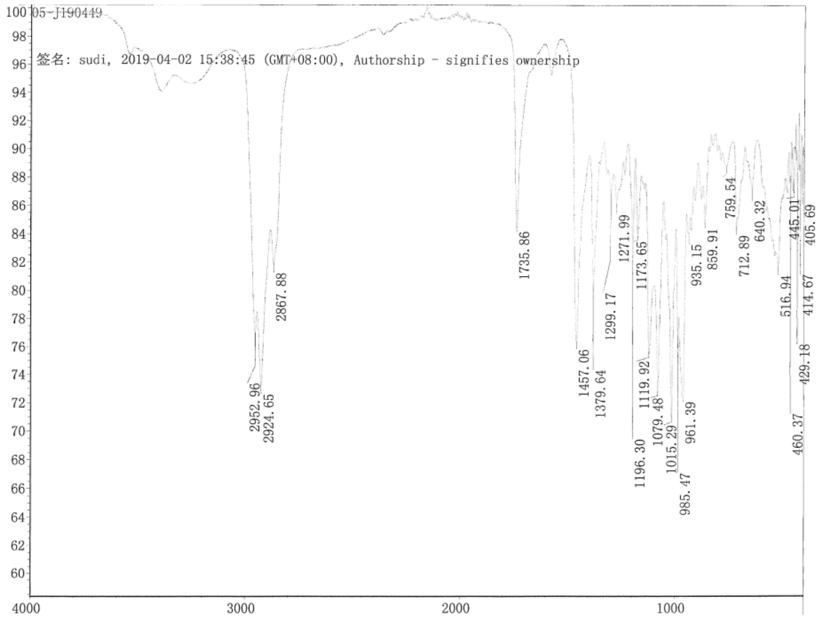

Figure S9. IR spectrum of compound **1** （in EtOH）

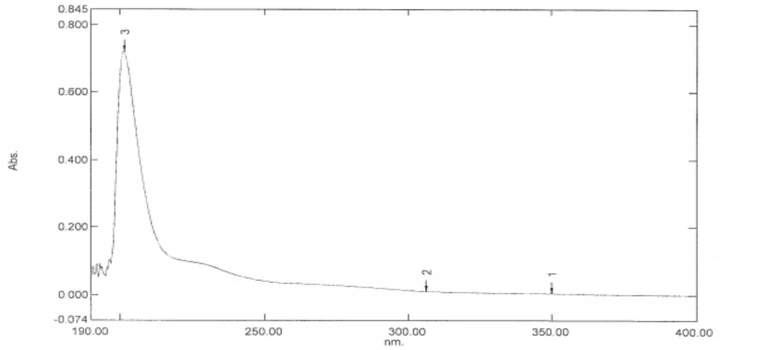

[Measurement Properties]
Wavelength Range (nm.): 190.00 to 400.00
Scan Speed: Fast
Sampling Interval: 0.2
Auto Sampling Interval: Enabled
Scan Mode: Single

[Instrument Properties]
Instrument Type: UV-2600 Series
Measuring Mode: Absorbance
Slit Width: 1.0
Accumulation time: 1.0 sec.
Light Source Change Wavelength: 360.0 nm
Detector Unit: Direct
S/R Exchange: Normal
Stair Correction: ON

[Attachment Properties]
Attachment: None

[Operation]
Threshold: 0.0010000
Points: 4
InterPolate: Disabled
Average: Disabled

[Sample Preparation Properties]
Weight: 25.04mg
Volume: 25ml
Dilution: 0.5ml-100ml
Path Length:
Additional Information:

No.	P/V	Wavelength	Abs.	Description
1	⊕	350.00	0.005	
2	⊕	306.20	0.011	
3	⊕	201.60	0.721	

Figure S10. UV spectrum of compound **1** (in EtOH)

**Sample Name**	NF813B-17	**Position**	Vial 1	**Instrument Name**	Instrument 1	**User Name**	CAY-JSA2-04\y
**Inj Vol**	0.1	**InjPosition**		**SampleType**	Sample	**IRM Calibration Status**	Success
**Data Filename**	NF813B-17-2.d	**ACQ Method**	MS.m	**Comment**		**Acquired Time**	3/26/2019 1:4

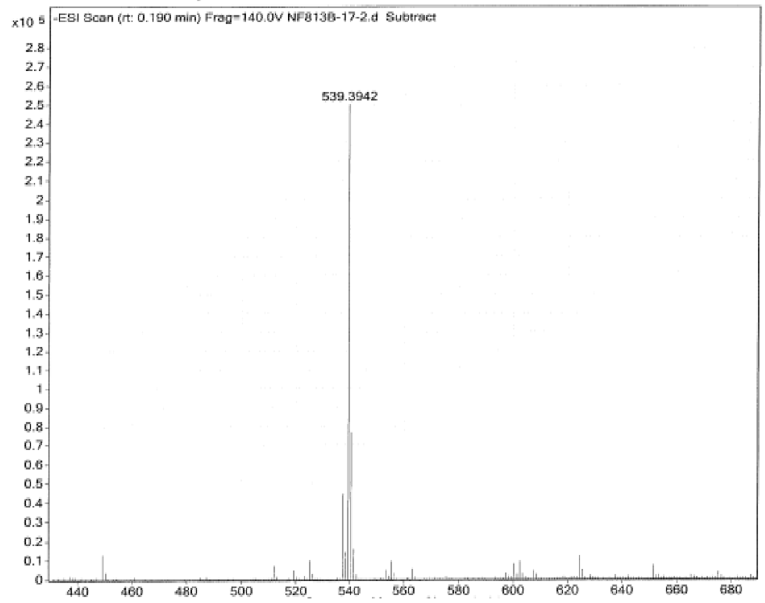

Figure S11. The HRESIMS spectrum of compound **1**

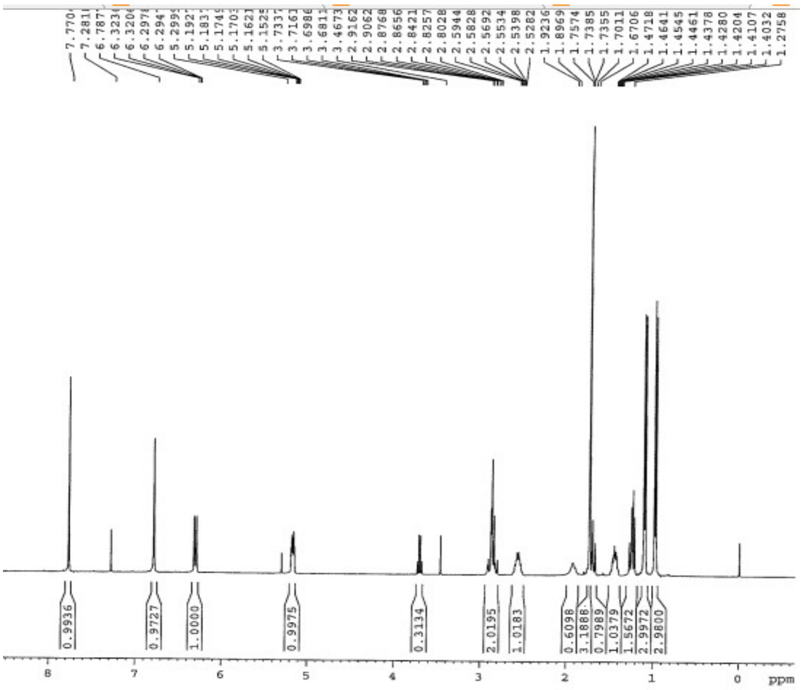

Figure S12. ^{1}H NMR (400 MHz) spectrum of compound **2** in $CDCl_3$

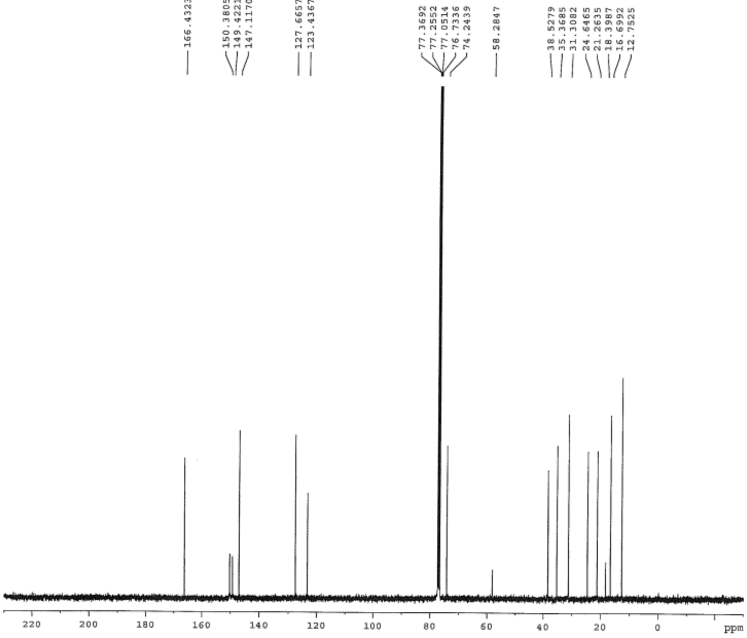

Figure S13. ^{13}C NMR (150 MHz) spectrum of compound **2** in $CDCl_3$

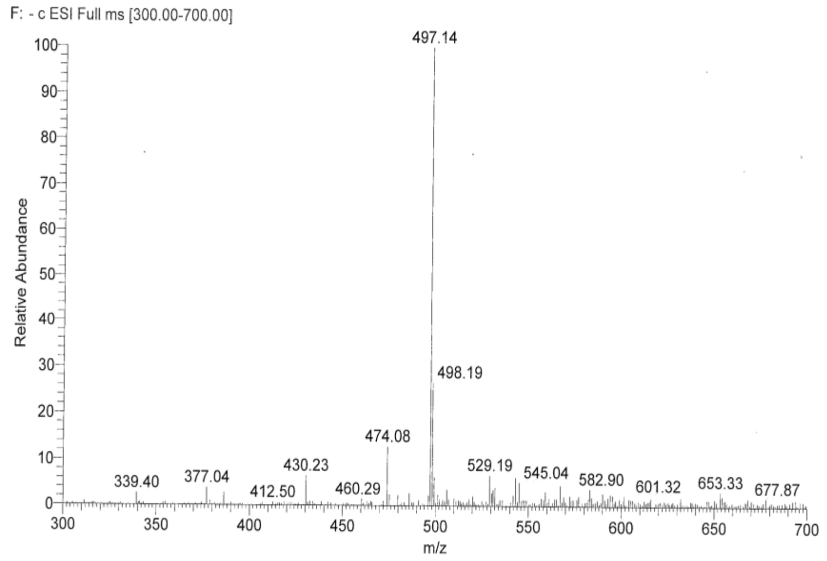

Figure S14. The ESI-MS spectrum of compound **2**

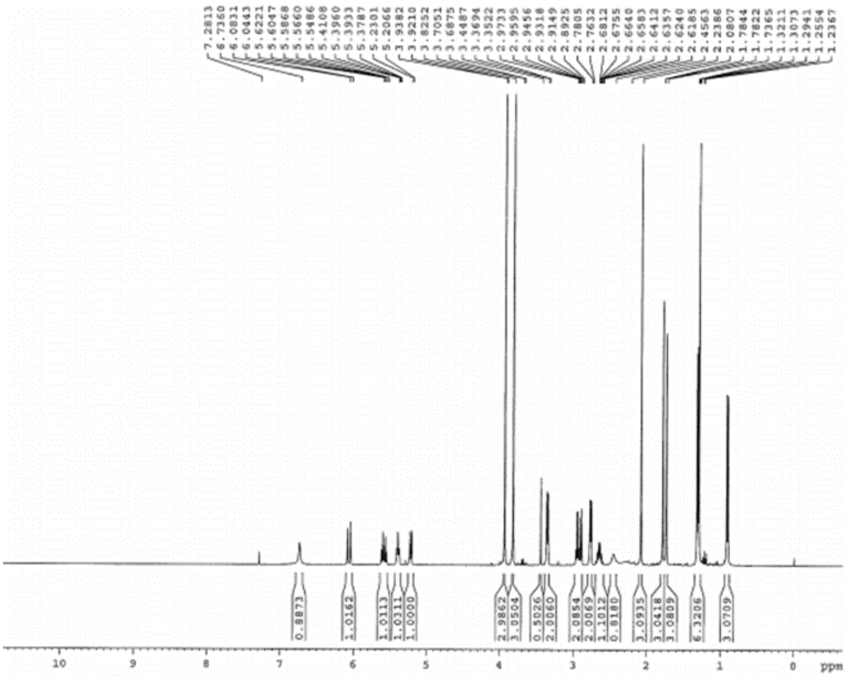

Figure S15. ^{1}H NMR (400 MHz) spectrum of compound **3** in $CDCl_3$

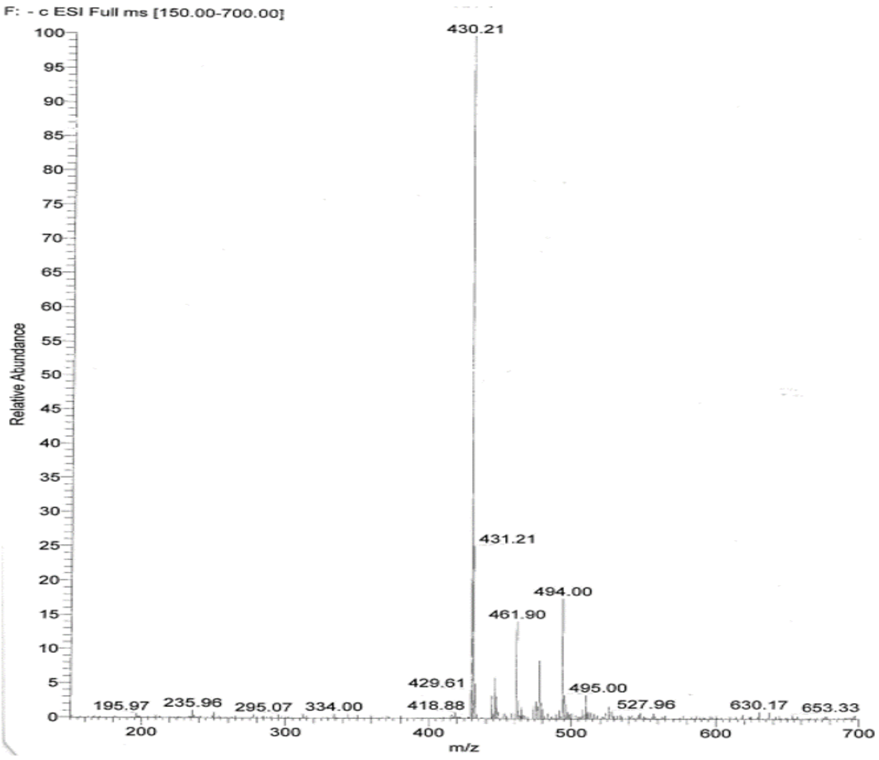

Figure S16. The ESI-MS spectrum of compound **3**

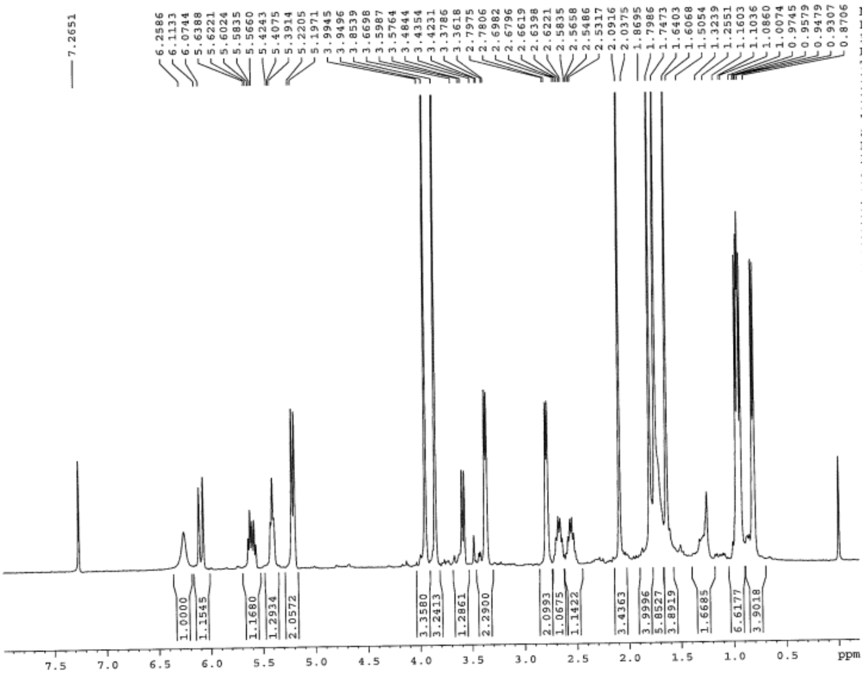

Figure S17. ^{1}H NMR (400 MHz) spectrum of compound **4** in $CDCl_3$

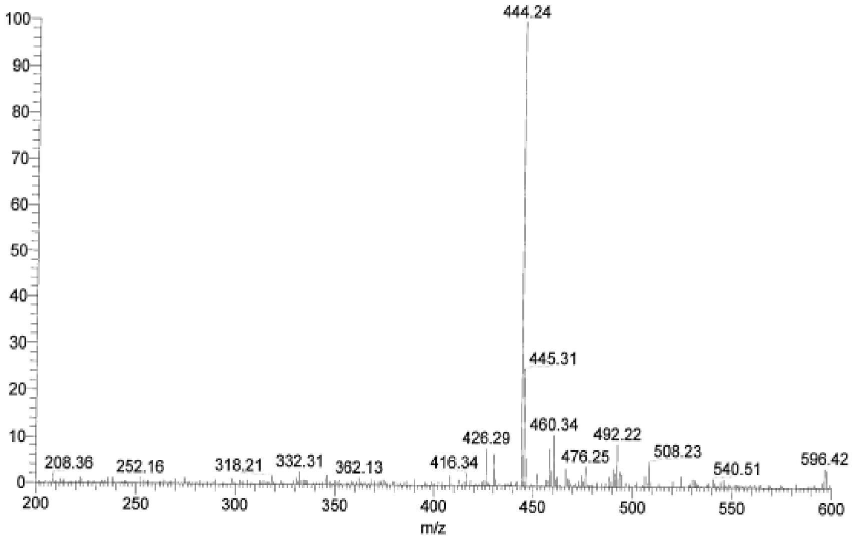

Figure S18. The ESI-MS spectrum of compound **4**

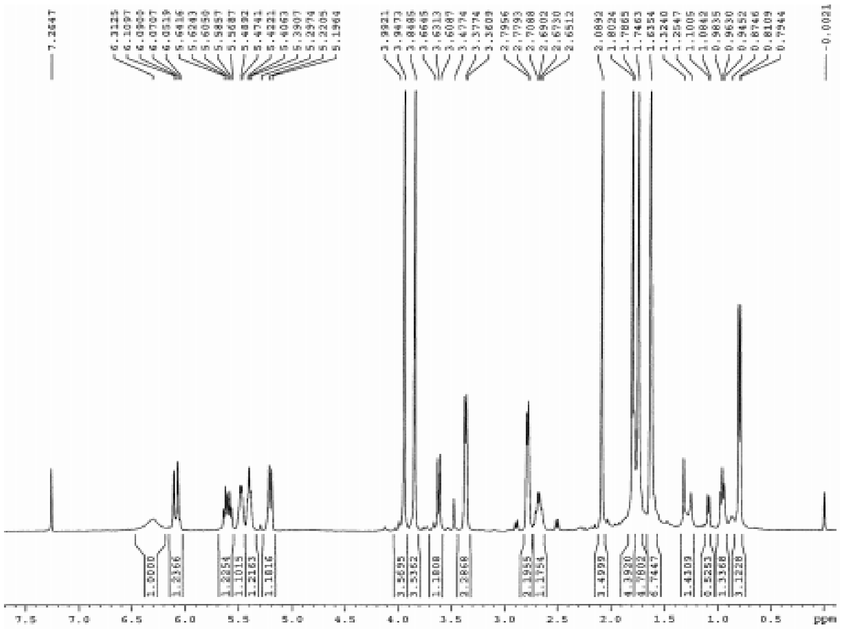

Figure S19. ^{1}H NMR (400 MHz) spectrum of compound **5** in $CDCl_3$

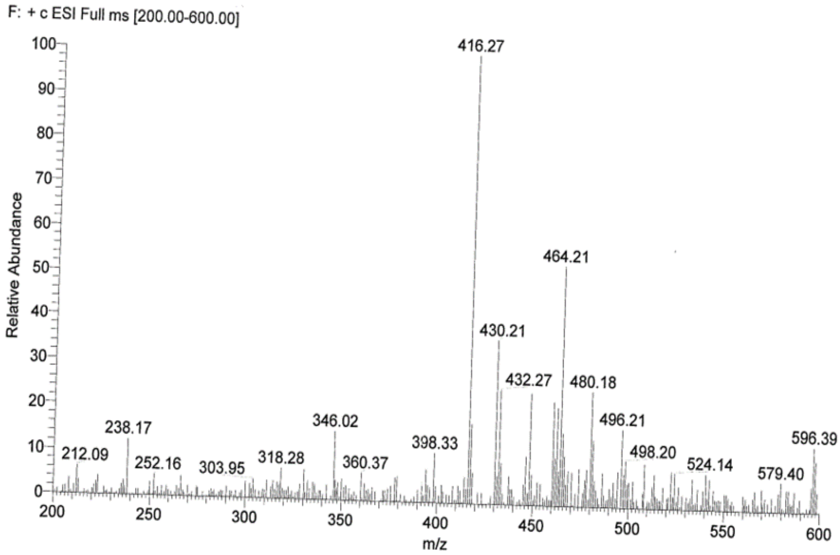

Figure S20. The ESI-MS spectrum of compound **5**

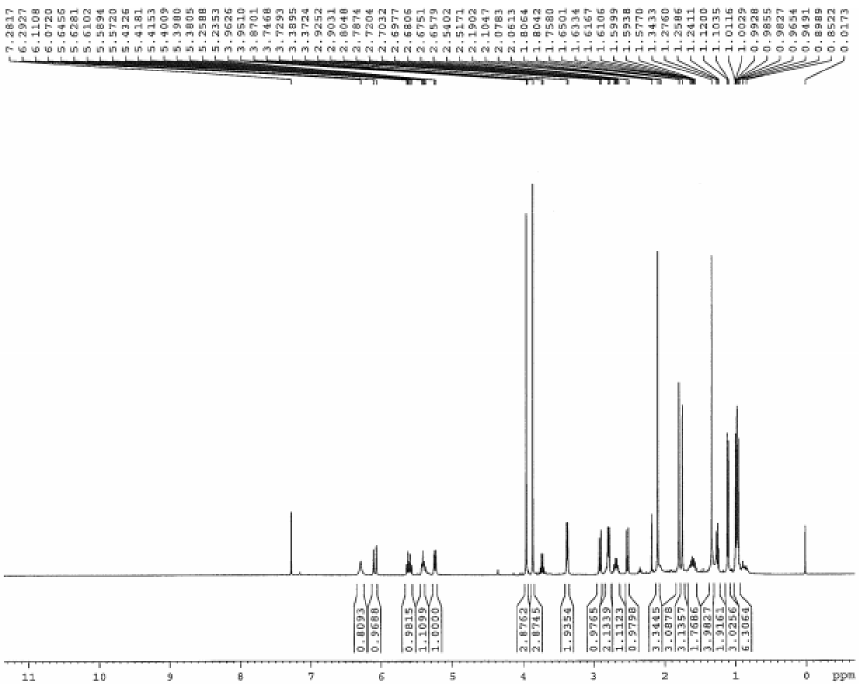

Figure S21. ^{1}H NMR (400 MHz) spectrum of compound **6** in $CDCl_3$

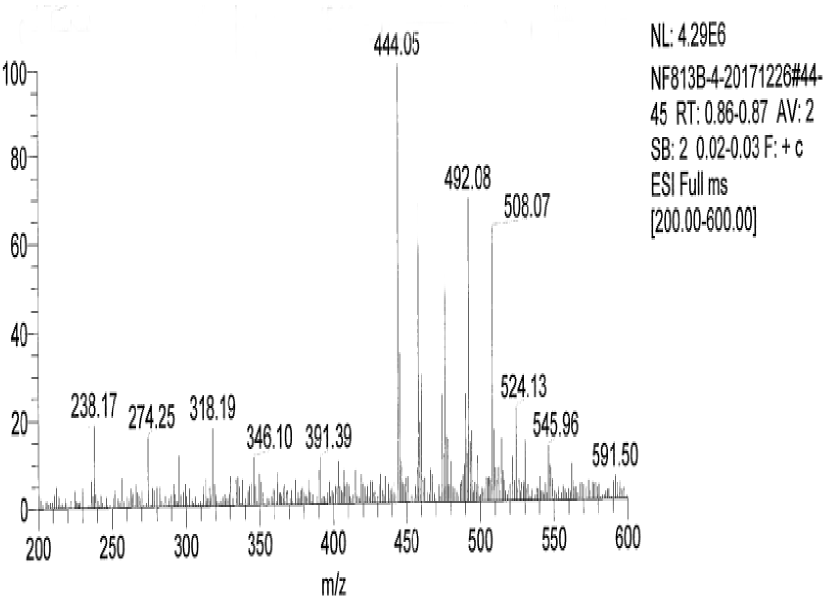

Figure S22. The ESI-MS spectrum of compound **6**

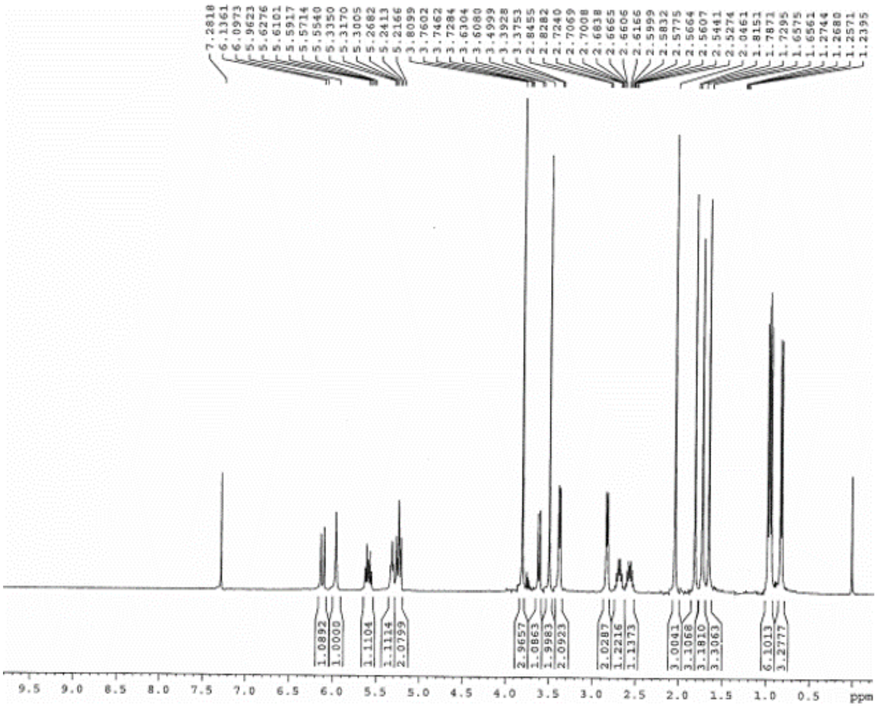

Figure S23. ^{1}H NMR (400 MHz) spectrum of compound **7** in $CDCl_3$

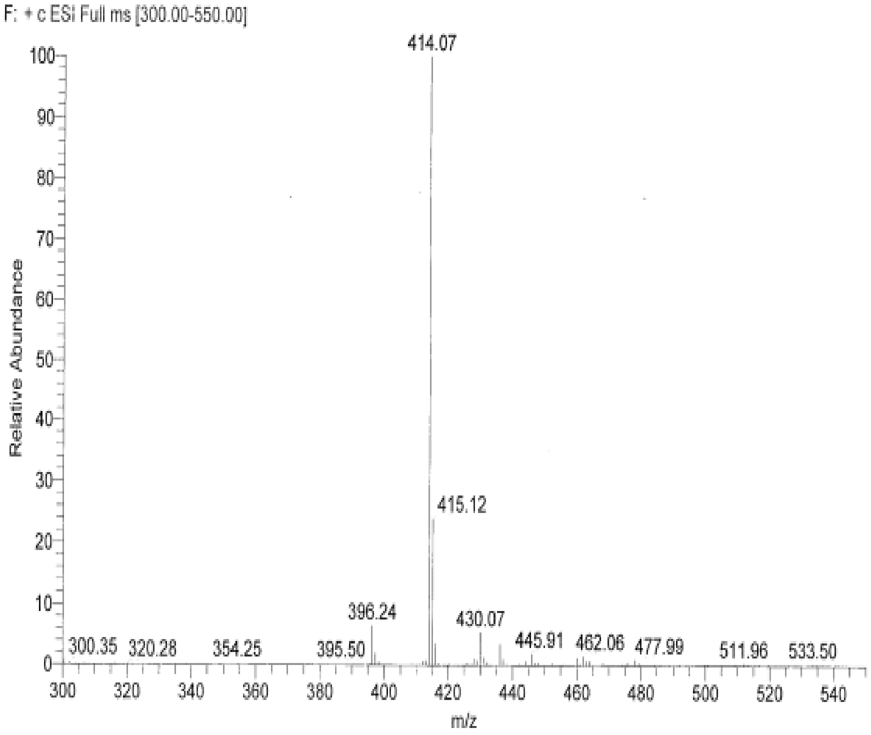

Figure S24. ESI-MS spectrum of compound of compound **7**

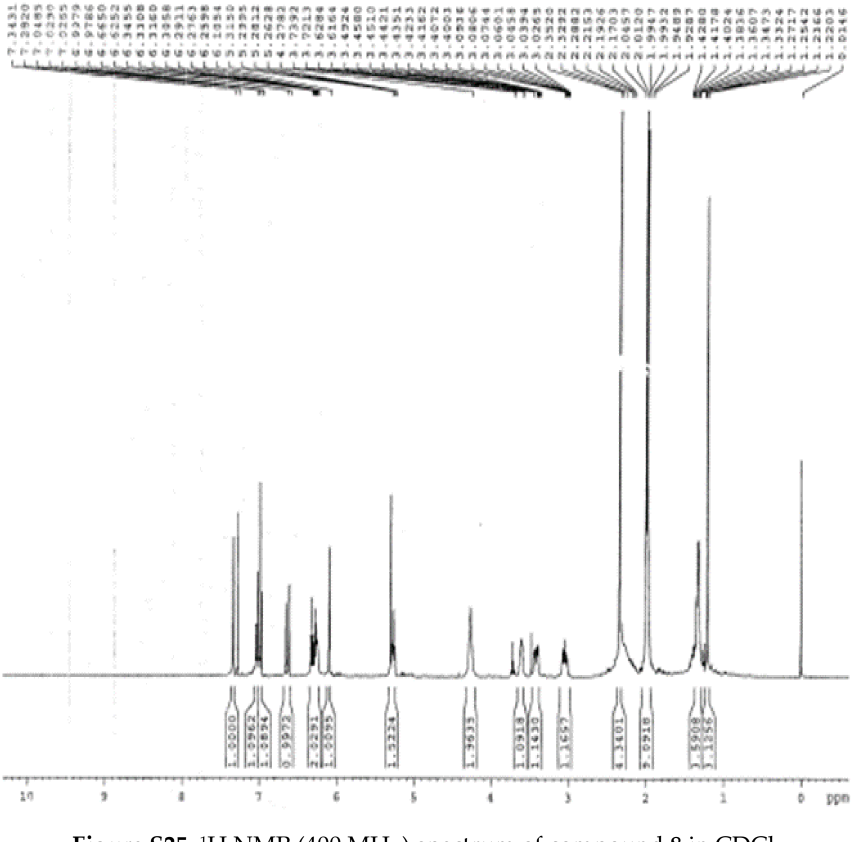

Figure S25. ^{1}H NMR (400 MHz) spectrum of compound **8** in $CDCl_3$

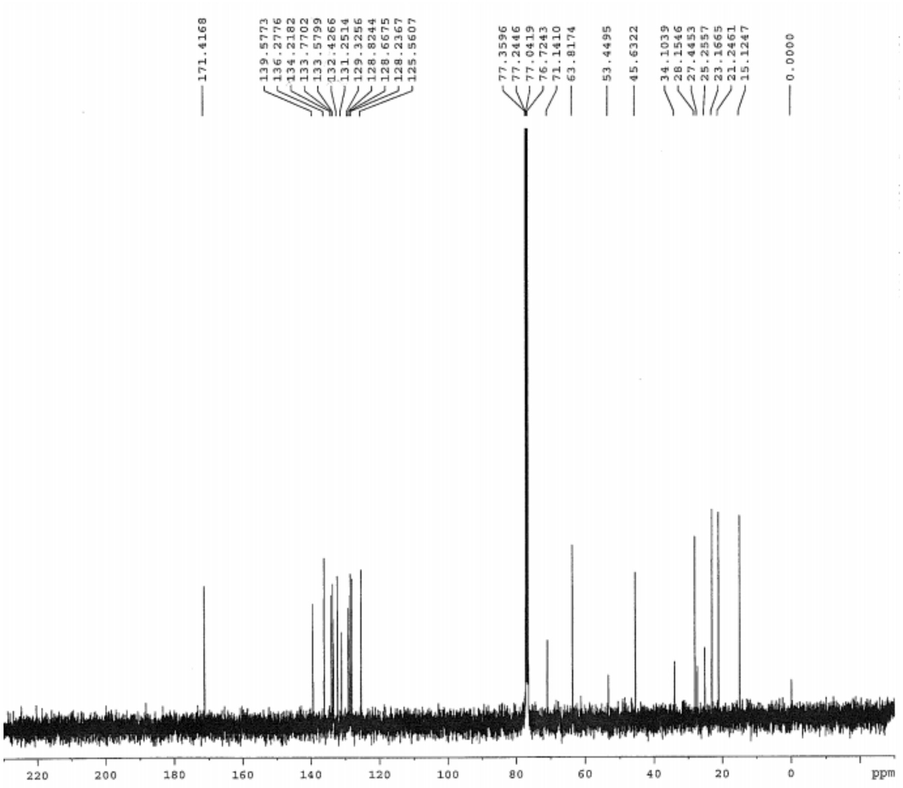

Figure S26. ^{13}C NMR (150 MHz) spectrum of compound **8** in $CDCl_3$

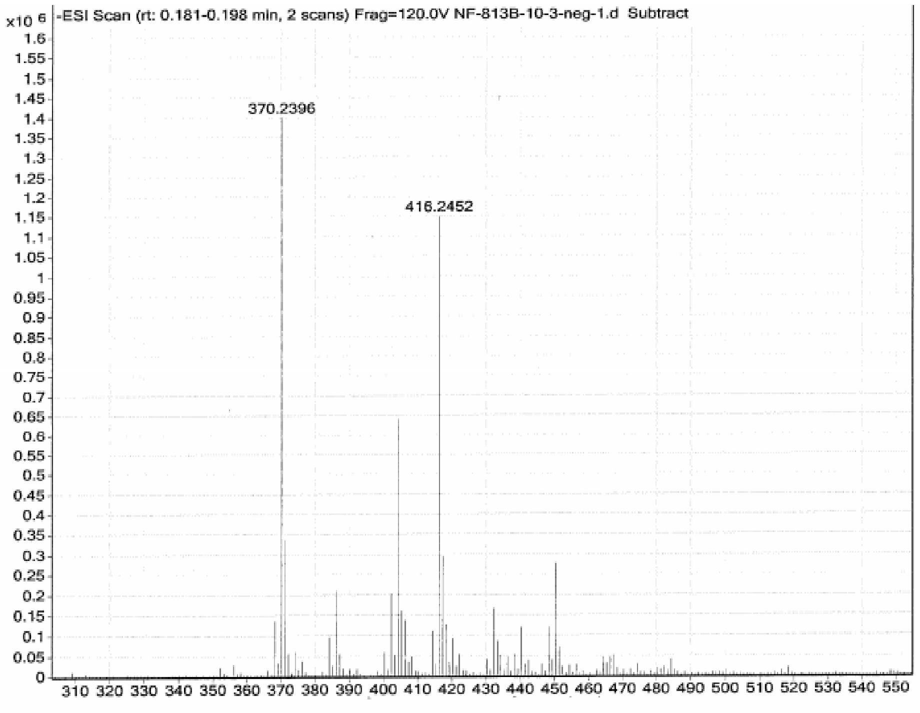

Figure S27. The HRESIMS spectrum of compound **8**
